# Irritable Bowel Syndrome: Manipulating the Endocannabinoid System as First-Line Treatment

**DOI:** 10.3389/fnins.2020.00371

**Published:** 2020-04-21

**Authors:** Viola Brugnatelli, Fabio Turco, Ulderico Freo, Gastone Zanette

**Affiliations:** ^1^Department of Neuroscience, University of Padua, Padua, Italy; ^2^Molecular Biology and Biochemistry Lab, Department of Neurogastroentherology, University of Naples Federico II, Naples, Italy; ^3^Department of Medicine, University of Padua, Padua, Italy

**Keywords:** IBS, endocannabinoid, CB1, CB2, TRPV1, THC, CBD, PEA

## Introduction

Irritable Bowel Syndrome (IBS) is a functional disorder characterized by abdominal pain, spasms, and altered bowel movements, either predominantly diarrhea (IBS-D), or predominantly constipation (IBS-C), or alternating between those states (Saha, 2014). In the Western world it affects the 10–15% of the population (Canavan et al., 2014). IBS represents a visceral hypersensitivity, with features of gastrointestinal (GI) allodynia and hyperalgesia. Considered a life-long condition, it is clear that significant gastrointestinal insults, such as food poisoning or antibiotic administration, may generate attacks that persist, often indefinitely. Attacks are associated with anxiety and depression, but controversy carries on to which incites the other (Saha, 2014). It is possible that some patients may develop a vicious cycle of worsening physical and psychological symptoms (Jones et al., 2013, 2017).

Currently, IBS sufferers are prescribed opioids, anticholinergics, and antidepressants, however with quite suboptimal results. Other compounds have been formulated to interact with serotoninergic circuitry, nevertheless these have been withdrawn from certain markets due to association with ischemic colitis (alosetron, cilansetron) and cardiovascular events (tegaserod), leaving, *de facto*, an urgent clinical need (Ford et al., 2014; Lexicomp Online, 2017).

The Endocannabinoid System (ECS) is known to modulate several functions, including mood, anxiety, and memory retrieval of traumatic events and it directly coordinates GI propulsion, secretion, inflammation, and nociception, providing a rationale for agents capable of interacting with the ECS as treatment candidates for IBS (Russo, 2016).

## Irritable Bowel Syndrome and the Endocannabinoid System

### Endocannabinoid System in the Bowel

The ECS is ubiquitously expressed in the human body and it actively controls gut homeostasis. The best characterized ECS receptors are the cannabinoid receptors 1 (CB1) and 2 (CB2) (Mackie, 2005).

CB1 has been found in intestinal epithelial and in the enteric nervous system (ENS) (Coutts and Izzo, 2004).

Physiologically, the activation of presynaptic CB1 attenuates large and small bowel muscle tone and inhibits GI motility, mainly by reducing the release of acetylcholine from enteric nerves and also by inhibiting all the components of the peristaltic reflex (Wright et al., 2005). Moreover, CB1 activation, via the purinergic system, inhibits spontaneous ileal contractions and modulate the activity of vagal neurotransmission, reducing intestinal peristalsis (DiPatrizio, 2016).

CB2 has been found on enteric neurons but it is predominantly expressed by intestinal immune cells (Izzo, 2007). Targeting intestinal CB2 decreases inflammation through the reduction of cytokine and chemokine production from activated immune cells (Wright et al., 2008). In pathophysiological conditions, CB2 controls intestinal motility (Wright et al., 2008) and its activation slows down gut transit (Mathison et al., 2004).

Bot1 and CB2 have been identified in the intestinal neuronal circuitry of the transmission of visceral pain and their activation reduce visceral sensation and nociception (Hohmann and Suplita, 2006).

N-arachidonoylethanolamine (anandamide, AEA) and 2-arachidonoyl glycerol (2-AG) are the best characterized endocannabinoids; they are synthesized from membrane phospholipids on demand: AEA is synthesized by N-acyl-phosphatidylethanolamine phospholipase D (NAPE-PLD); and 2-AG by diacylglycerol lipase (DAGL), then they are released and induce a local response by activating CB1 and/or CB2 receptors (the latter being involved mainly in pathophysiological conditions) (Izzo and Camilleri, 2008). These compounds are involved in the control of food intake and hunger (DiPatrizio, 2016; Lee et al., 2016). Specifically, AEA seems to regulate appetite and energy balance, while 2-AG may serve as a general hunger signal (Di Marzo and Matias, 2005; DiPatrizio, 2016). AEA, via CB2, plays also a pivotal role in maintaining immunological health in the gut (Acharya et al., 2017).

Subsequent to their activation, endocannabinoids are inactivated by reuptake from the degradative enzymes fatty acid amide hydrolase (FAAH), located in cells of the myenteric plexus and monoacylglycerol lipase (MAGL), present in the nerve cells and fibers throughout the muscle mucosal layers of the intestine (Di Marzo, 2006).

Inhibition of MAGL and FAAH in the gut significantly reduces experimental colitis in mice, through mechanisms that involve a rise in 2-AG or AEA levels, respectively, and the stimulation of both CB1 CB2 signaling (Massa et al., 2004; Sałaga et al., 2014; Vera and Fichna, 2017).

N-palmitoylethanolamine (PEA) and other N-acylethanolamides (NAEs) are also expressed in the gut (Izzo and Sharkey, 2010). NAEs are atypical endocannabionoids: their structures resemble the classical endocannabinoids and they are preferentially metabolized by FAAH, but they do not bind CB receptors (Izzo and Sharkey, 2010; Ahn et al., 2014). NAEs, especially PEA, are involved in the control of various functions, including food intake, neuroprotection, nociception, and inflammation (Suardíaz et al., 2007; Ahn et al., 2014; Lowin et al., 2015).

Other components of the ECS are the transient receptor potential (TRP) channels, such as TRPV1, TRM8, and others (Storozhuk and Zholos, 2018). These receptors, widely expressed throughout the digestive tract, are involved in numerous processes: taste, chemo- and mechanosensation, thermoregulation, pain and hyperalgesia, mucosal function, gut homeostasis, and control of motility, amongst others (Kaneko and Szallasi, 2014).

GPR55, another potential cannabinoid receptor, seems to be also implicated in gut motility. Its inhibition reduce motility in mice and this effect was reversed by cannabidiol (CBD), but not by CB1 or CB2 receptor antagonists (Li et al., 2013).

The ECS is also an important modulator of the gut-brain axis. In the gut, receptors of the ECS (especially TRPs) are involved in sensory transduction of a large number of external and noxious stimuli (Holzer, 2011). In the brain, the ECS controls nausea and vomiting, especially through CB1 receptors in the dorsal vagal complex of the brainstem, and stress-induced alterations of the ECS have been linked to altered visceral sensations (Sharkey and Wiley, 2016).

The main role of ECS in the GI tract is controlling intestinal hyper-contractility. Moreover, it modulates visceral sensations, intestinal inflammation and gut–brain communications, all functions that appear to be dysregulated in IBS.

### IBS and Endocannabinoid Deficiency

Clinical Endocannabinoid Deficiency (CED) has been confirmed as a plausible feature in a series of difficult-to-characterize psychosomatic pathologies, which display hyperalgesia, anxiety, and depression (Russo, 2004, 2016); Migraine, fibromyalgia and IBS fall in this category, often showing comorbidity in the three diagnosis (Nicolodi and Sicuteri, 1996; Sperber et al., 1999; Peres et al., 2001). CED occurs either as a congenital disorder, or as a result of epigenetic changes.

IBS subtypes exhibit distinct variations of the ECS tone. IBS-D patients show genetic alterations affecting endocannabinoid metabolism, variants of the CNR1 and FAAH genes, and lower levels of Oleoylethanolamine (OEA) and PEA compared to healthy subjects (Fichna et al., 2013). Specifically, the CNRI rs806378 CT/TT genotype shows a significant association with colonic transit in IBS-D (Camilleri et al., 2013). Conversely, IBS-C patients show levels of OEA higher than healthy volunteers, and reduced levels of FAAH mRNA in intestinal tissues (Fichna et al., 2013).

Some of these changes may occur as the result of chronic stress, which profoundly impacts the ECS: it silences the Cnr1 gene promoter via an increased methylation by DNA (cytosine-5)-methyltransferase 1, but it also activates the Trpv1 promoter via acetylation (Hong et al., 2015). This results in reduced levels of CB1 and increased levels of TRPV1 in the sensory neurons localized in the pelvic organs, including the colon, which is a feature of visceral pain, as later discussed (Fichna et al., 2013).

Stress in the early-life stage is also an important contributor to IBS development and it is associated with epigenetic changes that lead to visceral hypersensitivity (Moloney et al., 2015). Maternal deprivation increases the expression of the endocannabinoid genes Cnr1, Cnr2a, Cnr2b, and GPR55 in the frontal cortex of male rats, whereas in female rats, increased expression was reported only in the hippocampus, a difference that may underline the prevalence of IBS in the female population (Marco et al., 2014). The relevance of pediatric stress in IBS is supported by the fact that infantile colitis, characterized by visceral sensitivity and dysphoria and resistant to most pharmacotherapies, seem to be offset by the endocannabinoids present in maternal milk, reason for it is hypothesized that this condition may also be a CED (Russo, 2004). Taken these data together, genetic polymorphisms and alterations in gene expression are associated with disturbances in GI motility and sensation, supporting the pathophysiologic significance of alterations in the ECS in the gut (Moloney et al., 2015).

## Utilizing ECS-Modulating Agents for IBS

### ECS-Modulating Agents

Gut health devoid of pain and maintenance of balanced body weight seems to require a complex interplay between diet, enteric flora, and endocannabinoid balance (Clarke et al., 2012; Russo, 2016). Oral administration of *Lactobacillus acidophilus NCFM* induce a direct increase of the cannabinoid receptors CNR2 mRNA (Rousseaux et al., 2007). This result corresponded with an enhancement of morphine analgesic effect in rats, which was inhibited by administration of the CB2 antagonist, AM-630 (Rousseaux et al., 2007). Cannabinoids may also directly alter the microfloral balance, as underscored by the finding that THC affected the F*irmicutes:Bacteroidetes* ratio in obese mice, preventing their weight gain despite a high-fat diet (Cluny et al., 2015).

The interaction of the microbiome–gut–brain axis is highly dependent on hypothalamic–pituitary–adrenal (HPA) stress modulation, which is dysregulated in IBS patients (Chang et al., 2009). The ECS regulates basal and circadian HPA axis activation (Patel et al., 2004; Liedhegner et al., 2014), and these changes relate to the differences in visceral sensation that feature in IBS (Gschossmann et al., 2001). Linkage of the cannabinoid–vanilloid pathway to the HPA axis has been demonstrated by experiments monitoring rats inoculated with corticosterone, mimicking chronic stress, which developed visceral hyperalgesia (Hong et al., 2011); moreover, as also shown by another stressed rat model (Hong et al., 2009), the levels of AEA and the expression and phosphorylation of TRPV1 increased in the animals, whilst CB1 expression decreased in lumbosacral primary afferent neurons localized in the colon, but not in those innervating the lower extremities (Hong et al., 2009, 2011). AEA is an endogenous agonist at both CB1 and TRPV1 (McPartland et al., 2007), receptors that co-localize in nociceptive primary sensory neurons (Ahluwalia et al., 2000). Activation of CB1 inhibits nociception, whereas agonism at TRPV1 increases pain perception (Malik et al., 2015). Treatment of stressed rats with the CB1 agonist WIN 55,212-2 or the TRPV1 antagonist capsazepine prevented visceral hyperalgesia (Hong et al., 2009). Similar data have been observed in biopsies from IBS sufferers, which show a 3.5-fold elevation in TRPV1-immunoreactive nerve fibers (Akbar et al., 2008).

Considering this evidences, it has been posited that chronic stress causes down-regulation or loss of CB1, activation of the HPA stress response, anxiety, and induces visceral hyperalgesia that involve region-specific changes in endovanilloid and endocannabinoid pathways in sensory neurons innervating the pelvic viscera (Morena et al., 2016). Thus, a rationale exists for the use of compounds that boost AEA and PEA levels and desensitize TRPV1, to treat hypersensitivity and pain in IBS. While some authors have encouraged the use of the phytocannabinoid cannabidiol (CBD), no clinical trials have tested this hypothesis (Russo, 2004; Pandey et al., 2020). CBD may be an useful therapeutic intervention as it desensitizes TRPV1 and inhibits PEA and AEA hydrolysis and uptake (Bisogno et al., 2001).

Targeting endocannabinoid-degrading enzymes to increase AEA may be an interesting model (Sakin et al., 2015), given their role in the tonic disinhibition of periaqueductal gray region of the brainstem to promote analgesia and chronic stress-induced anxiety (Lau et al., 2014; Sakin et al., 2015). A dual FAAH and COX inhibitor has been shown to increase AEA and PEA levels, reducing features of colitis in mice (Sasso et al., 2015).

### Clinical Trials With ECS-Acting Agents

Despite the numerous lines of evidence showing the involvement of ECS in the regulation of IBS features and the promising data from pre-clinical studies, few clinical trials tested the effect of ECS-modulating agents in IBS.

On the other hand, ECS alteration in IBS patients has been clearly documented.

As ECS is known to decrease motility, effects of dronabinol, a non-selective agonist of the cannabinoid receptors, have been tested on IBS patients (Wong et al., 2011). In a 2011 clinical trial, dronabinol reduced fasting colonic motility in all IBS-D patients, particularly those carrying the CB1 receptor polymorphism rs806378 (Wong et al., 2011). Another clinical study carried out a few years later, failed to replicate these results, obtaining only modest delay in motility, maybe for differences in methods (manometry vs. radioscintigraphy) and the lower number of patients enrolled (Wong et al., 2012). Dronabinol can also improve visceral sensitivity and colonic relaxation, as showed in a double-blind, placebo-controlled trial (Esfandyari et al., 2007).

As mentioned before, Fichna et al. showed that lower PEA levels are associated with cramping abdominal pain (Fichna et al., 2013). A randomized placebo-controlled multicenter study assessing the efficacy of PEA in IBS, revealed that PEA may be an useful tool for pain management in this condition (Barbara et al., 2014).

Since visceral hypersensitivity is linked to an increase in Ts regard, a 2011 pilot study found that ingesting capsaicin-containing enteric-coated pills desensitized TRPV1 and decreased the intensity of abdominal pain and bloating in IBS patients vs. placebo (Bortolotti and Porta, 2011). Another study confirmed that TRPV1 desensitization reduced visceral hypersensitivity, symptoms, and abdominal pain (Wouters et al., 2016).

Menthol-induced analgesia and pain relief is mediated mainly by TRPM8 (Liu et al., 2013). This is the rationale for various trials that analyzed the efficacy of peppermint oil (containing menthol) in IBS. Even with some limitations mainly due to the delivery system of peppermint oil in the digestive tract, it turned out an effective treatment capable of improving IBS symptoms, especially abdominal pain, even in children suffering IBS (Kline et al., 2001; Cappello et al., 2007; Merat et al., 2010; Cash et al., 2016).

## Conclusions

Although the pathophysiology of IBS remains unclear, targeting the ECS may represent a promising strategy to modulate gut motility, visceral hyperalgesia, low-grade intestinal inflammation, and gut–brain axis alteration, all features that may improve IBS symptoms onset. It is also evident that both an IBS-diet (Wouters et al., 2016) and a stress-relief practice are required to boost the beneficial effects of any of the agents suggested.

**Figure 1:**
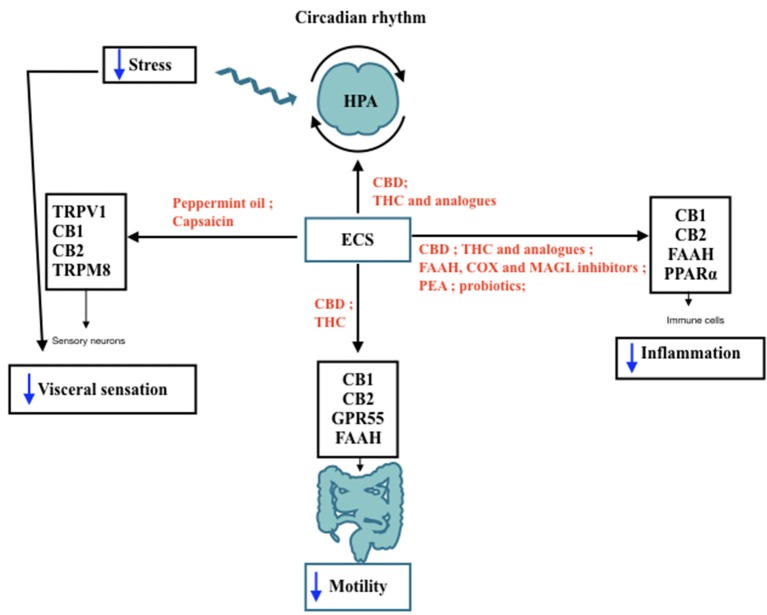
IBS features modulated by ECS. Schematic representation of the Endocannabinoid System (ECS) involvement in lBS features and its interaction with hypothalamic-pituitary-adrenal (HPA) axis throughout day/night. The black arrows indicate the receptors and the target sites controlled by ECS components. In red are shown the agents capable of modulating ECS activities that may be useful to improve IBS symptoms, such as motility, visceral pain, and low-grade inflammation. Blue arrows indicate a decrease in the functions targeted by ECS stimulation. TRPV I, Transient receptor potential vanilloid I; CB I, Cannabinoid Receptor I; CB2: Cannabinoid Receptor 2; TRPM8, Transient receptor potential melastatin 8; CBD, cannabidiol; THC, tetrahydrocannabinol; FAAH, fatty acid amide hydrolase; COX, cyclooxigenase; MAGL, monoacyl glycerol lipase; PEA, palmitoylethanolamide; PPARa: Peroxisome proliferator-activated receptor a.

In light of this, agents capable of modulating the ECS may provide a strategy worth attempting even first line treatment for IBS patients (Figure 1). This is due to the fact that compounds such as PEA, CBD and peppermint oil display a very large safety profile and have been proving beneficial to improve IBS symptoms (Halford et al., 2018); PEA, peppermint oil, THC and its synthetic analogs may be recommended in IBS patients to improve abdominal spasms, cramps and visceral pain. THC and CBD may alter ECS-driven response to the pathology. However, there is still a wide gap in the current understanding of IBS mechanism and in the use of cannabis containing both CBD and THC as potential therapy, which can only be bridged by randomized clinical trials.

## Author Contributions

VB and FT contributed to conception and design of the study and wrote sections of the manuscript. VB wrote the first draft of the manuscript. All authors contributed to manuscript revision, read, and approved the submitted version.

## Conflict of Interest

The authors declare that the research was conducted in the absence of any commercial or financial relationships that could be construed as a potential conflict of interest.
